# Economic burden of avoidable blindness due to diabetic macular edema in Ecuador

**DOI:** 10.3389/fpubh.2025.1476932

**Published:** 2025-07-14

**Authors:** Ruth Lucio, Juan Antonio Teran, Paula Luque

**Affiliations:** ^1^Sureste Research Group in Health Economics and Health Technology Assessment, Quito, Ecuador; ^2^Roche Sociedad Argentina de Investigación Clínica (SAIC), Buenos Aires, Argentina

**Keywords:** economic burden, diabetic retinopathy, diabetic macular edema, blindness, cost comparison, Ecuador, disability adjusted life years, productivity costs

## Abstract

**Introduction:**

In Ecuador, the economic burden of diabetes-related visual impairment has not been studied cohesively. More evidence—that takes into account lost productivity, direct costs, and intangible costs—is required to support public policies that prevent and treat diabetes-related visual impairment.

**Objective:**

The purpose of this research is to estimate the economic burden of avoidable blindness due to diabetic macular edema in Ecuador.

**Methods:**

Costs were estimated in a one-year, retrospective, cost-of-illness study focusing on two groups of people. The first group contains those that have become blind because of a lack of timely medical treatment for diabetic retinopathy (DR) and diabetic macular edema (DME). The second group contains those that could avoid blindness by receiving timely treatment. Productivity costs are costs associated with lost ability to work due to death, and with impaired (or lost) ability to work or to engage in leisure activities due to morbidity. Direct costs include direct healthcare costs and direct non-healthcare costs, i.e., costs incurred by patients and caregivers in the complete treatment of the disease. Intangible costs are a conceptual cost-compound which takes into account psychological aspects of disease such as anxiety/distress, and stigmatization.

**Results:**

In the year 2023, treating blindness caused by diabetic retinopathy and diabetic macular edema represents a yearly expense of USD 259.7 million—92.4% can be attributed to productivity costs. Preventing blindness caused by diabetic retinopathy and diabetic macular edema represents a yearly expense of USD 108.5 million—73.8% corresponds to costs of resources used in interventions for health promotion and disease prevention, only 26.2% corresponds to medical treatment. The difference between these two scenarios is 151.2 million; in other words, the cost of treating a person that has become disabled costs USD 33,518.98 more per year than trying to prevent the disability.

**Conclusion:**

The cost to society of providing timely treatment for diabetic retinopathy and diabetic macular edema is significantly less than the cost of supporting a person that has become blind due to lack of treatment. Thus, it would be prudent to invest in public policies that prevent and treat diabetes-related visual impairment.

## 1 Introduction

Diabetic macular edema (DME) is the thickening of the retina caused by accumulation of intraretinal fluid; it is a major cause of blindness in people of productive age and a direct consequence of diabetic retinopathy (DR), which is the most common microvascular complication of diabetes mellitus.

The global prevalence of diabetes mellitus has tripled in the past 20 years and is expected to increase. Worldwide, approximately 422 million people have diabetes mellitus; of these, 146 million (34.6%) have some form of diabetic retinopathy. In the group with DR, approximately 47 million have a type of DR that is vision threatening, and 4.3 million already have moderate-to-severe visual impairment or blindness (1–8). It is estimated that, worldwide, approximately 5.5% of people with diabetes have DME (9).

In Ecuador, in 2023, approximately 526.7 thousand people have diabetes mellitus and, applying the worldwide prevalence of DR (34.6%), we estimate that 182.2 thousand people have some form of diabetic retinopathy. We also estimate that in the group with DR almost 60,000 persons have a type of DR that is vision threatening; and that more than 4,500 persons already have blindness due to DR (7%) (8, 10). This estimate is validated with data from the national registry of disabilities which estimates that Ecuador has 61,799 blind persons (11). From this blind population, it is estimated that between 4.8% (3,000) and 7.3% (4,511) are blind due to avoidable DR/DME (12–14).

Diabetes mellitus is the third largest cause of death in Ecuador, making it and its consequences—namely visual impairment—a significant public health issue (15). But the economic burden of diabetes-related visual impairment to Ecuadorian society has not been studied cohesively and more evidence—that takes into account lost productivity, direct costs, and intangible costs—is required to support public policies that prevent and treat diabetes-related visual impairment (economic burden refers to the financial strain placed on individuals, families, and society due to costs associated with healthcare, social services, and other necessities for dependent populations).

The purpose of this research is to *estimate the economic burden of avoidable blindness due to diabetic macular edema in Ecuador*.

**Figure 1:**
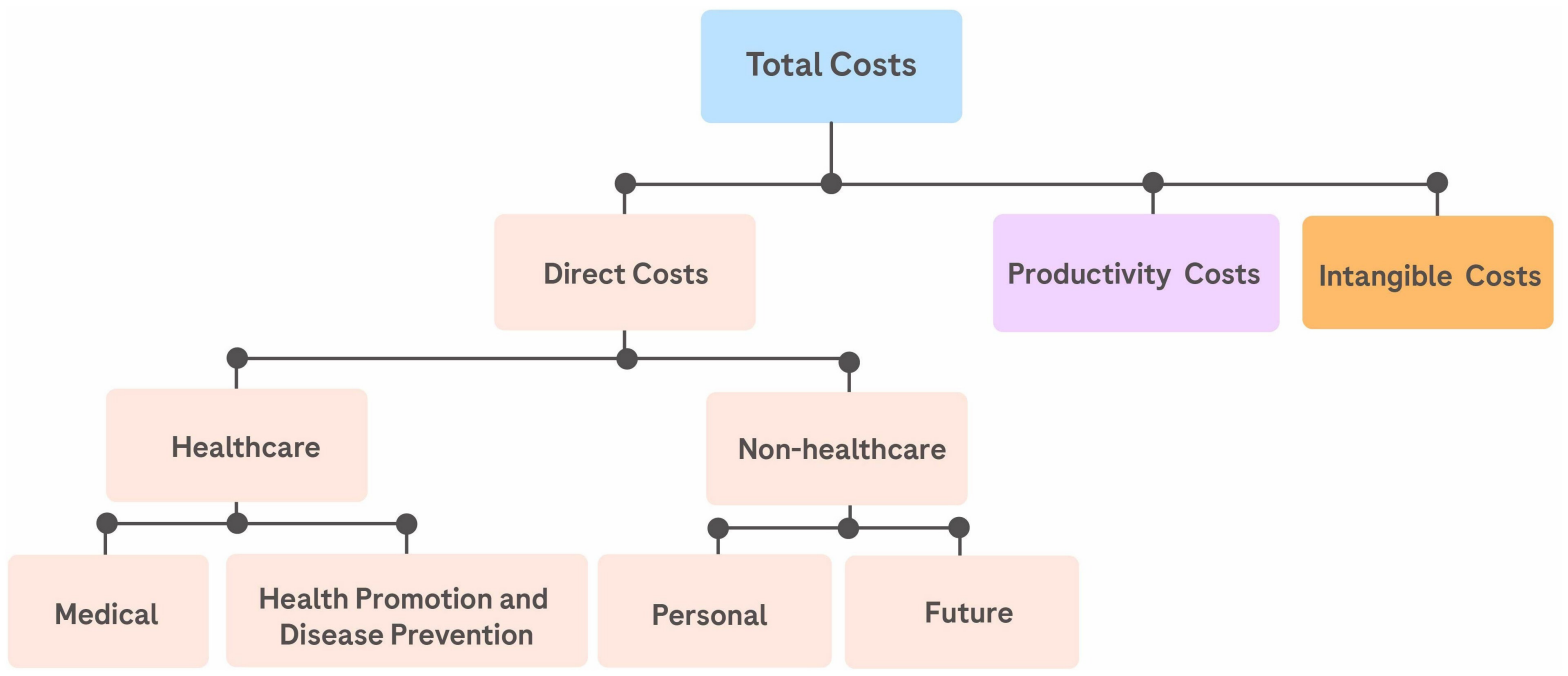
Cost typology: disentangling health-related costs.

Here we compare the annual costs of providing timely treatment to diabetic retinopathy and diabetic macular edema patients to the costs undertaken by Ecuadorian society in the support of people that have become visually impaired due to a lack of timely treatment. [Direct costs, productivity costs, and intangible costs are considered; which is to say that we move beyond a pure direct-costs perspective and consider factors crucial to reflect the realities of Ecuadorian healthcare (Figure 1).] And we find that the cost to society of providing timely treatment for diabetic retinopathy and diabetic macular edema is significantly less than the cost of supporting a person that has become blind due to lack of treatment.

## 2 Methods

The research on costs associated with providing timely treatment for diabetic retinopathy and diabetic macular edema (vs. the cost of supporting blindness sufferers) was conducted as a 1-year, retrospective, cost-of-illness study (16). Our research focuses on two groups: group A, people that have become blind because of a lack of timely treatment for diabetic retinopathy (DR) and diabetic macular edema (DME); and group B, people with vision threatening DR or DME that could avoid blindness by receiving timely medical treatment. Group A corresponds to the 4,500 blind persons referred to in the introduction section; group B corresponds to the almost 60,000 persons with a type of DR that is vision threatening.

To calculate the total cost of DME for our studied groups we consider direct costs, productivity costs, and intangible costs; which is to say that we move beyond a pure direct-costs perspective and consider factors crucial to reflect the realities of Ecuadorian healthcare. Among these factors are: lost productivity; Ecuadorian government resources allocated to social protection policies for the visually impaired; and all costs which are not directly related to medical attention but are attached to managing a health condition on a day-to-day basis.

### 2.1 Direct costs

Direct Costs are classified as *direct healthcare costs* and *direct non-healthcare costs*.

#### 2.1.1 Direct healthcare costs

Direct healthcare costs (DHC) include costs related to medical treatment (medication, imaging, laboratory services, and the cost of treatment itself) and costs of resources used in interventions for health promotion and disease prevention. To estimate DHC, a *bottom-up* approach is used: we estimate the average cost of a treatment or intervention and multiply that by either disease prevalence or by the number of people participating in a health-promotion activity (17–19).

##### 2.1.1.1 Costs related to medical treatment

The values calculated are a product of the number of people that receive an intervention and the cost of the intervention for one patient (these figures are adjusted considering that treatment can be received in the public or private sector). Note that in many cases the number of people receiving an intervention is 4,511, which is an amount equal to the number of people in group A. This is because we assume that *the number of people known to have become blind having received no prevention or treatment*, should be similar to *the number of people who can avoid blindness by receiving timely medical treatment*.

##### 2.1.1.2 Costs of interventions for health promotion and disease prevention

As it pertains to group A, the term *interventions for health promotion and disease prevention* refers to interventions that deal with blindness and, also, to interventions for general health—i.e., we first consider interventions designed for blind people and, when these are not directly available, we consider interventions directed toward managing disability in general and we adjust for our target population. As it pertains to group B, *interventions for health promotion and disease prevention* refers to those which would result in detection and timely treatment of DR/DME.

#### 2.1.2 Direct non-healthcare costs

Direct non-healthcare costs include direct personal costs, i.e., costs incurred by patients and caregivers in the complete treatment of the disease, such as transportation, meals, lodging, and time (18). We value time using the *human capital method* (with respect to wages earned in normal employment, and taking into account waiting time, commuting time, and time spent on completion of a session of medical treatment). Direct non-healthcare costs also include future costs: resources allocated by society to increase life-expectancy or life-years. We value future costs by valuing the resources that the Ecuadorian government allocates to support blind people and the betterment of collective ophthalmological health (20).

##### 2.1.2.1 Direct personal costs for group A

To estimate direct personal costs for group A, the following is considered: (i) Blind people require assistance from a caregiver when receiving health interventions (this includes assistance during transportation, wait, and treatment) (21). (ii) We distinguish between *resident* and *non-resident* patients and caregivers. Residents are those who live in the city where the treatment facility is located (approximately 80%), non-residents are those who live outside of this city. (iii) Non-resident patients and caregivers have different transportation and food costs than residents and, additionally, they have lodging costs. (iv) To adjust for different income levels, patients and caregivers should be grouped by income brackets (22). (v) Caregivers are assumed to be in the same income bracket as the patients in their care.

The income brackets used for our categorization are: *Legally mandated minimum salary (LMMS) for 40 h of work per week*: USD 582.75 per month. *Low income*: USD 209 per month. *Median income*: USD 668 per month. *High income*: USD 1695 per month.

### 2.2 Productivity costs

Productivity costs are the costs associated with impaired (or lost) ability to work or to engage in leisure activities due to morbidity (17, 20) (In this study death is not considered, as it is not a direct result of DME).

Productivity costs include three elements: (i) cost of time for patients and caregivers (P&C), (ii) cost of lost-income assistance payments made by the government of Ecuador to those unable to work, and (iii) cost of disability benefits provided by the government of Ecuador. (i) Cost of time is estimated for the patient as what the patient would earn in normal working conditions (with no visual impairment). In cases where the patient is fully blind, we have also estimated cost of time for the caregiver in terms of opportunity cost (the cost of giving up regular employment, as the caregiver must give time and energy to support the visually impaired person on a day-to-day basis).

(iii) Cost of disability benefits is estimated by analyzing the effects of the Ecuadorian *Organic Disabilities Law* (*LOD* ^1^). This is an “umbrella” legislation that attempts to encompass all aspects of disability and regulate all institutions, public and private. Because of how far-reaching this legislation is, we had to not only estimate the cost of benefits directly mandated by law but also many of the benefits that occur as side effect of the letter of the law.

We note that:

- Ecuador is a country with a *Gini index* of 0.45; this indicates income inequality and warrants considering income by quintile. The following data for average annual income by quintile is used: Quintile 1 has an Average Annual Income of USD 1,848; Quintile 2 has USD 4,275; Quintile 3 has USD 13,248; Quintile 4 has USD 26,640; Quintile 5 has USD 44,700 (22, 23).
- Lost productivity is weighted by disability-adjusted life years. We assume the following: lost productivity of diabetic retinopathy patients is weighted 0.031; lost productivity of visually impaired patients is weighted 0.184; lost productivity of adults of working age that have completely stopped working because of blindness is weighted 1; lost productivity for the caregiver is weighted 0.5 (used in cases where the patient is fully blind) (24, 25).

### 2.3 Intangible costs

Intangible costs are a conceptual cost-compound used to input a cost on pain and suffering which takes into account psychological aspects of disease such as anxiety/distress, and stigmatization (19, 26). Central to our conception of intangible costs is the consideration that social concerns surrounding a disease create a pressure which, in turn, creates a response that is economically quantifiable—at least insofar as the shadow price of the different responses can be calculated. In Ecuador responses to social pressures from a disease are seen in, at least, three contexts: (i) payment for the education of personnel for mental health support, viz. psychiatrists, to guide patients and families through the emotional burdens of living with a disease or disability, (ii) the creation and maintenance of a bureaucracy to deal with customer dissatisfaction, and (iii) the work of NGOs.

## 3 Results

### 3.1 Direct healthcare costs related to medical treatment for group A

For the year 2023, for group A, the total costs related to medical treatment are estimated at approximately USD 10.9 million.

This figure includes: (i) The cost of blindness hospitalizations due to DR and collateral conditions (27) (USD 215,884.19); calculated as the product of hospitalization costs (found in the *Ecuadorian National Health System price list*) (28), and the average number of hospitalizations due to blindness and collateral conditions, as registered by the Ecuadorian National Health System (29). (ii) The total annual cost of outpatient visits required by group A (USD 647,732.69); calculated as the product of the number of average annual visits required by one patient, and the cost of a consultation. (iii) The total annual cost of clinical rehabilitation therapy (USD 10,079,378.40); calculated as the product of the number of people in group A (4,511), and the annual cost of therapy for a patient/family from group A (USD 2,234.40).

### 3.2 Direct healthcare costs related to medical treatment for group B

For the year 2023, for group B, the total costs related to receiving timely medical treatment are estimated at approximately USD 28.4 million.

This figure includes: (i) The total annual cost of ophthalmologic exams (USD 1,478,488.18); product of a population of 4,511 with 70% of patients receiving treatment in the public sector and 30% in the private sector, and an annual per-patient cost of USD 331.04 in the public sector and USD 320 in the private sector. (ii) The total annual cost of laboratory exams (USD 602,344.81); product of a population of 4,511 with 70% of patients receiving treatment in the public sector and 30% in the private sector, and an annual per-patient cost of USD 141.28 in the public sector and USD 115.44 in the private sector. (iii) The total annual cost of laser photocoagulation (USD 143,242.93); product of the number patients who would receive this treatment (5.5% of 4,511), and a per-eye cost of USD 552.64 in the public sector and USD 635 in the private sector [again, a 70/30 proportion]. (iv) The annual cost of anti-VEGF injection therapy (USD 24,155,268.23) was estimated using the per-person cost of *Faricimab* (USD 4,864.56). The total annual cost of vitrectomy (USD 55,273.28) was estimated considering costs of location/equipment usage, health professionals, and necessary medication—we calculated a composite unit priced at USD 2,450.6. (v) The total annual cost of intravitreal steroids (USD 528,895.58) was estimated considering costs of location/equipment usage, health professionals, and necessary medication—we calculated a composite unit priced at USD 4,324.5 for the public sector, and USD 5,055.16 for the private sector. (vi) the total annual cost of visual rehabilitation (USD 1,457,666.50)—which is used when other treatments have failed (in approximately 36% of cases)—is estimated with an annual per-patient cost of USD 897.6.

### 3.3 Costs of interventions for health promotion and disease prevention for group A

For the year 2023, the total cost of resources used in interventions for health promotion and disease prevention for group A is estimated at approximately USD 4.4 million.

This figure includes: (i) Publicly-funded social-work interventions to facilitate blind-person adaptation to society (USD 48,233.54). (ii) Disability aids provided by the Ministry of Health (USD 170,000). (iii) Expenses from the *MIES-PFD* project^2^ (USD 3.94 million). (iv) Awareness campaigns for inclusive public spaces organized by Ecuadorian municipalities (USD 12,000). (v) Privately-funded social-work interventions (USD 96,467.07)—these are varied activities meant to improve how society interacts with disabled people. (vi) Disability aids and legal aid provided by blind-person associations (USD 67,698). (vii) Canine support services provided by NGOs (USD 60,000)—we estimate a yearly average of 5 trained dogs reaching the population at a cost of USD 12,000 each. (vii) Optical equipment manufacturing (USD 41,000). (viii) Insurance payments related to ophtalmological health and accidents (USD 8,500). [These last two elements, vii and vii, refer to specific entries of the same name in the *United Nations System of National Accounts* (30)].

94% of these resources are financed publicly,^3^ so the primary source of information to estimate these costs was the *General Budget of the State* (31). The remaining resources, 6%, are financed privately, with most funding coming from families and blind-person associations.^4^

### 3.4 Costs of interventions for health promotion and disease prevention for group B

For the year 2023, for group B, the total cost of interventions for health promotion and disease prevention is estimated at approximately USD 80 million.

This figure includes: (i) Home visits (USD 61,771.10) [The Ecuadorian *Ministry of Health* organizes comprehensive health campaigns that reach approximately 200,000 people per year. (32) These campaigns include home visits that, in practicality, serve as a mechanism for early identification of diseases and guidance of the population to receive further, specialized, treatment. In our study, we value the portion of home visits that reach people with diabetes mellitus, i.e., those at risk for DR/DME.] (ii) The total annual cost of laboratory exams (USD 2,264,121.11); product of the annual per-patient cost of laboratory exams, and the number of people that require this service. (iii) The total annual cost of ophthalmologic exams (USD 5,305,171.67); product of the annual per-patient cost of ophthalmologic, exams and the number of people that require this service. (iv) Varied interventions for health promotion and disease prevention organized by the *Ministry of Health*^5^ (USD 222,895.39). (v) The cost of household expenditures in optical devices (USD 72,081,000). [This element refers to a specific entry from the *United Nations System of National Accounts* (30)].

### 3.5 Direct non-healthcare costs

#### 3.5.1 Direct personal costs for group A

The annual direct personal costs for group A, for *resident* patients and caregivers, vary from USD 102.4 to USD 655.88, depending on income bracket. The annual direct personal costs for group A, for *non-resident* patients and caregivers, vary from USD 1,214.9 to USD 4,093.11, depending on income bracket. Table 1 summarizes our findings and aggregates totals for the entire population in the year 2023.

**Table 1:** Group A direct personal costs of health maintenance/repair.

**Income bracket**	**Resident P&C**	**Non-resident P&C**	**Total cost (USD)**
Bracket 1 (LMMS)	875,242.26	1,753,624.18	2,628,866.45
Bracket 2 (low income)	369,468.94	1,096,091.80	1,465,560.75
Bracket 3 (median income)	990,615.60	1,903,605.91	2,894,221.51
Bracket 4 (high income)	2,366,939.74	3,692,803.84	6,059,743.59

#### 3.5.2 Future costs for group A

For the year 2023, total future costs are estimated at approximately USD 230,000.

This figure includes: (i) The total annual cost of ophthalmologist and optometrist training which will be used to serve patients with DR/DME (USD 64,350); product of the average cost of professional training per ophthalmologic consultation for DR/DME patients (USD 14.27), and the number of people that have become blind because of a lack of timely treatment (4,511). (ii) Accessibility modifications (USD 65,585.30); this is the sum of various entries in the *General Budget of the State (GBS)* related to improving accessibility for blind people. However, the values found in the GBS are for the whole population of Ecuador and for all disabilities so they were scaled to the population relevant for our study. (iii) The costs of maintaining a bureaucracy to serve the blind (USD 99,816.30) were calculated by identifying which institutions are required^6^ to promote public policies and administrate resources needed to support people with disability^7^; and then valuing, for each institution, the portion of the budget used specifically for this task.

### 3.6 Productivity costs

Productivity costs are estimated at approximately USD 240 million.

This figure includes: (i) The cost of time for patients and caregivers (USD 164,213,438.61); product of average annual income by quintile, the number of people with ophthalmologic conditions (DR/DME, visual impairment, and blindness), and the cost of lost productivity. (ii) The cost of lost-income assistance payments (USD 13,226,330.90) made by the government of Ecuador to those unable to work; estimated considering cash transfers distributed by the government of Ecuador, and pension payments from the Social Security Administration of Ecuador. (iii) The cost of disability benefits (USD 62,530,449.70) to group A; estimated by attempting to account for all the different benefits that come as a direct or indirect result of the Ecuadorian *Organic Disabilities Law* (*LOD*) (33, 34) [we find that benefits are distributed mostly through the areas of economics/internal revenue (69.03%) and health (26.2%). Other benefits come through education (3.3%), tourism and others (1.29%), and social inclusion (0.17%)].

### 3.7 Intangible costs

For the year 2023, the intangible costs of becoming blind due to lack of treatment of DR/DME are estimated at approximately USD 1.2 million.

This figure includes: (i) Annual cost of education of personnel for mental health support to guide patients and families through the emotional burdens of living with a disease or disability (USD 6,087.00); product of the average cost of professional training per consultation for DR/DME patients, and the percentage of the DME-blind population which receives mental health support. (ii) The total annual cost of the creation and maintenance of a bureaucracy to deal with customer^8^ dissatisfaction (USD 857,142.86). This was estimated using a composite unit which considers the number of public employees who work in customer dissatisfaction, their pay, the cost to attend each customer, and the percentage of total complaints that can be attributed to group A. (iii) The total annual cost of the work of NGOs in the assistance of the blind (USD 355,650.00); product of the total number of people directly affected by blindness working in these NGOs and the annual cost of work time for each person, added to a basic overhead cost.

### 3.8 Total costs

*Treating* blindness caused by diabetic retinopathy and diabetic macular edema, in the year 2023, represents an expense of USD 259.7 million per year (36.45% financed by the government of Ecuador, and 63.55% by the private sector). From this total, 92.4% can be attributed to productivity costs, 7.1% to direct costs, and 0.5% to intangible costs. Still referring to the 259.7 million, 63.2% corresponds to the cost of lost work and opportunity costs, and 29.1% corresponds to the assistance payments and benefits distributed by the government (see Table 2).

**Table 2:** Total costs for group A.

**Costs for group A**	**Total (USD)**	**Public (USD)**	**Private (USD)**
**Direct costs**	**18,512,054**	**18,215,296**	**296,758**
*(i) Healthcare*	15,387,807.66	15,114,142.59	273,665.07
medical treatment	10,942,882.35	10,942,882.35	0.00
HP/DP	4,444,925.31	4,171,260.24	273,665.07
*(ii) Non-healthcare*	3,124,246.85	3,101,153.89	23,092.95
personal	2,894,221.51	2,894,221.51	0.00
future	230,025.34	206,932.38	23,092.95
**Productivity costs**	**239,984,814**	**75,600,1610**	**164,384,653**
*(i) Cost of time P&C*	164,228,138.11	0.00	164,228,138.11
*(ii) Cost of lost-income assistance payments*	13,226,226.72	13,069,711.40	156,515.32
disability insurance	233,604.96	77,089.64	156,515.32
cash transfer	12,992,621.76	12,992,621.76	0.00
*(iii) Cost of disability benefits*	62,530,449.70	62,530,449.70	0.00
**Intangible costs**	**1,218,880**	**863,230**	**355,650**
*(i) Education of personnel for mental health support*	6,087.29	6,087.29	0.00
*(ii) Bureaucracy for customer dissatisfaction*	857,142.86	857,142.86	0.00
*(iii) Work of NGOs*	355,650.00	0.00	355,650.00
**Total (USD)**	**259,715,749**	**94,678,687**	**165,037,061**

*Preventing* blindness caused by diabetic retinopathy and diabetic macular edema, in the year 2023, represents an expense of USD 108.5 million per year (20.9% financed by the government of Ecuador, and 79.1% by the private sector). From this total, 73.8% corresponds to costs of resources used in interventions for health promotion and disease prevention, and 26.2% corresponds to medical treatment (see Table 3).

**Table 3:** Total costs for group B.

**Costs for group B**	**Total (USD)**	**Public (USD)**	**Private (USD)**
**Direct costs**	**108,511,638.78**	**22,632,972.61**	**85,878,666.16**
*(i) Healthcare*	108,511,638.78	22,632,972.61	85,878,666.16
medical treatment	28,421,179.51	14,779,013.35	13,642,166.16
HP/DP	80,090,459.27	7,853,959.27	72,236,500.00
**Total (USD)**	**108,511,638.78**	**22,632,972.61**	**85,878,666.16**
public/private percentage of total		20.9%	79.1%

The difference between these two scenarios is 151.2 million; in other words, the cost of treating a person that has become disabled costs USD 33,518.98 more per year than trying to prevent the disability.

## 4 Discussion

Diabetes-related visual impairment has not been studied cohesively in terms of lost productivity, direct costs, or intangible costs to Ecuadorian society as a whole. Our study is guided by the tenet that analysis from a pure direct-costs perspective ignores various important factors and is not enough to integrate the academic research with the realities of Ecuadorian healthcare. To account for these factors, we look at direct costs, productivity costs, and intangible costs.

Regarding social inequality, although we've made an effort to adjust for differences in income and some of the differences related to rural/urban inequality—which is notable in Ecuador and Latin America—it is likely that our estimations are understated. More precisely, the additional cost of treating a person that has become disabled from DR/DME is *likely to be higher* than our estimated USD 33,518.98.

### 4.1 Implications

The largest provider and payer of health services in Ecuador is the state. The *Ministry of Health* is responsible for 61% of financing and 73% of facilities (35). But, after taking into account agreements with the private sector and the Social Security institute (which also provides health services), the state is actually responsible for almost 92% of health care in Ecuador (36).

This means in practicality that if the Ecuadorian Ministry of Health has not specifically considered a disease, and specifically allocated a portion of the budget to its prevention and treatment, the disease will be almost ignored in the country.

When considering and budgeting for a disease, those in charge of health policy face challenges such as:

- a lack of information regarding intangible costs to Ecuadorian society,
- inferring what portion of the state budget is already allocated to a disease (the official information available only gives values for large categories and makes no attempt at itemization),
- quantifying the direct effect that programs and interventions from other sectors have on managing the disease. (For example: economic aid to disability may be thought of, logically, as part of the cost of managing a disease. But because much of this aid is under the purview of the Ministry of Social Inclusion, it is considered a cost of “social inclusion” and there may be no *official* reason, or method, to include it as part of the healthcare budget).

And thus, it is rare to see comparisons between the costs of providing timely treatment and the costs undertaken by Ecuadorian society in the support of people that suffer from a fully-developed disease. And it is even more rare to see a multidimensional approach to costing—one that takes into account pain and suffering brought on by living with a disability.

### 4.2 Impact and future research

We believe that our study can have an impact as an example of how to calculate the intangible costs of a disease for countries that share similar characteristics to Ecuador (low income, fragmented health systems, and deficient and outdated information systems).

Our study can be seen as an application of two concepts. The first is that social concerns surrounding a disease ultimately create a response that is economically quantifiable; and it is applied by looking at government budget expenditures in sectors outside of health to identify programs and interventions that may impact the quality of life of those ailing from the disease and calculate total costs. The second considers the reality seen in the field that private institutions in Ecuador have better incentives to measure results and record accurate data than public institutions; and it is applied by gathering information from non-traditional sources such as blind-person associations—these sources may, practically, provide more relevant data than obvious sources such as the General Budget of the State. And these concepts can be applied in future research on the cost of lost productivity due to disabling disease.

With respect to the practical implications of our study, our results should be an incentive to start campaigns for early screening of the disease, at the very least. These campaigns would not be an exotic demand or an imposition on the budget, they would be coherent with programs for chronic disease that are already mandated and already budgeted-for.

In addition to this, we believe that our study raises questions about how different institutions in the Ecuadorian government can influence treatment of the same disease and still have no communication with, or consideration of, each other. This study is a good starting point to make a case for creating a permanent inter-institutional commission on avoidable blindness.

### 4.3 Limitations of the study

The biggest limitation we faced in this research has to do with scarcity of data specific to our topic, since most data in Ecuador is collected and published only for broad categories (i.e. there is available data for diabetes, general disability, and budget allocated to institutions as a whole but *not* for DR/DME, blindness as a disability, or the specific portion of the budget allocated to the disease). As an example, the Ecuadorian *General Budget of the State* does not have enough granularity to determine how the budget is spent per disease—and there is no obvious way to find this information, if it even exists.

Another limitation was faced when trying to determine the benefits promised by the law and expressing them as measurable units. This is a complex and not always successful task because it requires perusing legal and bureaucratic documents which are not as clear in Ecuador as they may be in more developed countries. This task requires seeking out information from those who have personal experience applying for and receiving these benefits; and this information, irrespective of its quality, comes piecemeal and may become outdated because of legal and political vagaries.

Yet another limitation has to do with a scarcity of literature that analyses costs comprehensively; while the realities of Ecuadorian healthcare—inequalities, inefficiencies, tacit agreements, and complicated legal background, among other problems—make it *necessary* to analyse costs comprehensively, fully accounting for productivity and intangible costs, and not rely solely on direct cost estimations to reach conclusions. Thus, many of the situations we came across had no precedent in the literature and required us to find ad-hoc ways of estimating and incorporating these factors. As an example, our estimation of intangible costs was heavily dependent on analyzing social response to a disease because this response is economically quantifiable—at least insofar as shadow prices can be calculated.

Despite limitations, this study is the first comprehensive research on the costs of diabetic macular edema for Ecuador. We expect that the methods of estimation used, although not ideal, can be considered as an example of how to deal with a situation that is very common and unlikely to improve anytime soon (i.e. legal ambiguity with regard to how benefits are distributed and how budget should be utilized, and unavailability of data). And thus, we hope that this study can serve as a reference for future studies dealing with the economic burden of disease, in Ecuador and Latin America.

## 5 Conclusion

In Ecuador, as of 2023, diabetic macular edema is a disease that places a significant and growing strain on society; its effects are felt not just financially but also as a decrease in the quality of life.

The annual cost of timely treatment (USD 1,809) is equivalent to 28% of the GDP produced by one person;^9^ and this treatment makes it possible, even likely, for a person to remain in or return to productive activity. On the other hand, the annual cost of benefits provided after blindness has developed (USD 57,569.63) is equivalent to approximately 900% of the GDP produced by one person.

So, even under conservative assumptions, the cost to society^10^ of providing timely treatment for diabetic retinopathy and diabetic macular edema is significantly less than the cost of supporting a person that has become blind due to lack of treatment.

We conclude that it would be prudent to invest in public policies that prevent and treat diabetes-related visual impairment.

## Data Availability

The original contributions presented in the study are included in the article/Supplementary material, further inquiries can be directed to the corresponding author.

## Footnotes

^1^This acronym is widely used in Ecuador and comes from the law's original name in Spanish: *Ley Organica de Discapacidades*.

^2^A treatment, prevention, and rehabilitation project from the *Ministry of Economic and Social Inclusion* directed to improve attention for people with disability.

^3^These resources are financed mostly through the *Ministry of Economic and Social Inclusion*, and the *Ministry of Health*.

^4^Most notable amongst these are FENCE: *Federación Nacional de Ciegos del Ecuador* [National Federation of the Blind of Ecuador] and FOAL: Fundación ONCE para la solidaridad con las personas ciegas de Amrica Latina (ONCE: Organizacion Nacional de Ciegos Espaoles) [FOAL: ONCE foundation for solidarity to the blind persons of Latin America (ONCE: National Organization of the Blind of Spain)].

^5^A variety of organizations (such as clubs for diabetes patients), activities (such as informational sessions, and physical activities), and campaigns (such as the “*Sights of Hope*”/“Miradas de Esperanza” campaign) were considered in estimating this value.

^6^By the Ecuadorian *Organic Disabilities Law*.

^7^Identification of institutional duties is, in itself, a non-trivial task which requires a separate effort.

^8^i.e., patients and caregivers.

^9^The annual Ecuadorian GDP per capita is USD 6,500.

^10^The burden of blindness caused by preventable DME falls mostly on the private sector—which includes patients and families—with 63.55%, while the Ecuadorian government takes on 36.45% of the burden.
