# Supplementary material for: Economic burden of avoidable blindness due to diabetic macular edema in Ecuador
Source: Front Public Health. 2025 Jul 14;13:1476932. doi: 10.3389/fpubh.2025.1476932 (PMC12301294; doi:10.3389/fpubh.2025.1476932)
Supplement: Supplementary file 1 [file Data_Sheet_1.pdf]

Supplementary Material: Economic Burden of Avoidable Blindness due to Diabetic Macular Edema in Ecuador

1 SUPPLEMENTARY TABLES AND FIGURES

	Resident P&C	Non-resident P&C	Total Cost (USD)
Bracket 1 (LMMS)	875,242.26	1,753,624.18	2,628,866.45
Bracket 2 (low income)	369,468.94	1,096,091.80	1,465,560.75
Bracket 3 (median income)	990,615.60	1,903,605.91	2,894,221.51
Bracket 4 (high income)	2,366,939.74	3,692,803.84	6,059,743.59

Table S1. Group A direct personal costs of health maintenance/repair

Costs for group A	Total (USD)	Public (USD)	Private (USD)
**Direct Costs**	**18,512,054**	**18,215,296**	**296,758**
(i) Healthcare	15,387,807.66	15,114,142.59	273,665.07
medical treatment	10,942,882.35	10,942,882.35	0.00
HP/DP	4,444,925.31	4,171,260.24	273,665.07
(ii) Non-healthcare	3,124,246.85	3,101,153.89	23,092.95
personal	2,894,221.51	2,894,221.51	0.00
future	230,025.34	206,932.38	23,092.95
**Productivity Costs**	**239,984,814**	**75,600,1610**	**164,384,653**
(i) Cost of time P&C	164,228,138.11	0.00	164,228,138.11
(ii) Cost of lost-income assistance payments	13,226,226.72	13,069,711.40	156,515.32
disability insurance	233,604.96	77,089.64	156,515.32
cash transfer	12,992,621.76	12,992,621.76	0.00
(iii) Cost of disability benefits	62,530,449.70	62,530,449.70	0.00
**Intangible Costs**	**1,218,880**	**863,230**	**355,650**
(i) Education of personnel for mental health support	6,087.29	6,087.29	0.00
(ii) Bureaucracy for customer dissatisfaction	857,142.86	857,142.86	0.00
(iii) Work of NGOs	355,650.00	0.00	355,650.00
**Total (USD)**	**259,715,749**	**94,678,687**	**165,037,061**

Table S2. Total Costs for Group A

Costs for group B	Total (USD)	Public (USD)	Private (USD)
**Direct Costs**	**108,511,638.78**	**22,632,972.61**	**85,878,666.16**
(i) Healthcare	108,511,638.78	22,632,972.61	85,878,666.16
medical treatment	28,421,179.51	14,779,013.35	13,642,166.16
HP/DP	80,090,459.27	7,853,959.27	72,236,500.00
**Total (USD)**	**108,511,638.78**	**22,632,972.61**	**85,878,666.16**
public/private percentage of total		20.9%	79.1%

Table S3. Total Costs for Group B

Treatment (Group A)	Annual Cost (USD)
Blindness hospitalizations due to DR (ICD-10:H360)	10,941.57
Blindness hospitalizations due to collateral causes	204,942.69
Outpatient visits required by group A	647,732.69
Clinical rehabilitation therapy	10,079,378.40
**Total**	**10,942,995.35**

Table S4. Group A annual medical treatment costs

Treatment (Group B)	Annual Cost (USD)
Ophthalmologic exams	1,478,488.18
Laboratory exams	602,344.81
Laser photocoagulation	143,242.93
Anti-VEGF injection therapy	24,155,268.23
Vitrectomy	55,273.28
Intravitreal steroids	528,895.58
Visual rehabilitation	1,457,666.50
**Total**	**28,421,179.51**

Table S5. Group B annual medical treatment costs

Intervention for HP/DP (Group A)	Annual Cost (USD)
Publicly-funded social-work interventions	48,233.54
Disability aids provided by the *Ministry of Health*	170,000.00
MIES-PFD project	3,941,026.70
Awareness campaigns for inclusive public spaces	12,000.00
Privately-funded social-work interventions	96,467.07
Disability aids provided by blind-person associations	67,698.00
Canine support services provided by NGOs	60,000.00
Optical equipment manufacturing	41,000.00
Insurance payments related to opht. health and accidents	8,500.00
**Total**	**4,444,925.31**

Table S6. Group A annual costs of interventions for health promotion and disease prevention (HP/DP)

Intervention for HP/DP (Group B)	Annual Cost (USD)
Home visits (part of comprehensive health campaigns)	61,771.10
Laboratory exams	2,264,121.11
Ophthalmologic exams	5,305,171.67
Preventive counseling and/or risk factor reduction interventions	86,846.87
Local-government campaigns	3,500.00
Diabetes-club establishment and maintenance	4,342.34
Supplements and information on nutrition	128,206.18
Household expenditures in optical devices	72,081,000
Insurance payments related to ophtalmological health and accidents	8,500.00
Manufacture of chemical and pharmaceutical products	143,000.00
Manufacture of optical instruments	4,000.00
**Total**	**80,090,459.27**

Table S7. Group B annual costs of interventions for health promotion and disease prevention (HP/DP)

Group A expenses incurred in the process of receiving health interventions		

Logistic expenses		
	**Cost for resident P&C (USD)**	**Cost for non-resident P&C (USD)**
Food consumed in trip	N/A	183.36
Transportation	24.00	384.00
Lodging	N/A	240.00
**Total**	**24.00**	**807.36**

Cost Of Time		
	**Time for resident P&C**	**Time for non-resident P&C**
Transportation	36 hours	288 hours
Waiting for attention	12 hours	12 hours
Consultation and intervention	12 hours	12 hours
Total time	60 hours	312 hours
**Cost of time for bracket 1**	$218.53	$1,136.36
**Cost of time for bracket 2**	$78.38	$407.55
**Cost of time for bracket 3**	$250.50	$1,302.60
**Cost of time for bracket 4**	$631.88	$3,285.75

**Total Expenses Incurred (Direct Personal Costs)**		
	**Cost for resident P&C (USD)**	**Cost for non-resident P&C (USD)**
Bracket 1 (LMMS)	242.53	1,943.72
Bracket 2 (low income)	102.38	1,214.91
Bracket 3 (median income)	274.50	2,109.96
Bracket 4 (high income)	655.88	4,093.11

Table S8. Group A expenses incurred in the process of receiving health interventions

	**Future Costs**	**Public**	**Private**	**Total Cost (USD)**
32.	Ophthal. and optometrist training	41,257.78	23,092.95	64,350.73
33.	Accessibility modifications	65,585.30	—	65,585.30
34.	Bureaucracy to serve the blind	99,816.30	—	99,816.30
	**Total**	**206,932.38**	**23,092.95**	**230,025.34**

Table S9. Future Costs

**Institution**	**Budget for disabilities (USD)**
Consejo Nacional para la Igualdad de Discapacidades (CONADIS)	901,500
Ministerios de Inclusión Social	1.42 mill
Ministerio de Salud Pública del Ecuador	1.03 mill
Ministerio de Educación	6.44 mill
Servicio de Rentas Internas	180,000
Registro Civil del Ecuador	451,000
**Total**	**10,426,500**

Table S10. Disability Budget Per Institution

**Productivity Costs**	**Annual Cost (USD)**
Cost of time (for P&C)	164,213,438.61
Cost of lost-income assistance payments	13,226,330.90
Cost of disability benefits	62,530,449.70
**Total**	**239,970,219.20**

Table S11. Productivity Costs

Intangible Costs	Annual Cost (USD)
Education of personnel for mental-health support	6,087.00
Bureaucracy to deal with customer dissatisfaction	857,142.86
Work of NGOs	355,650.00
**Total**	**1,218,880.14**

Table S12. Intangible Costs

1.1 Figures

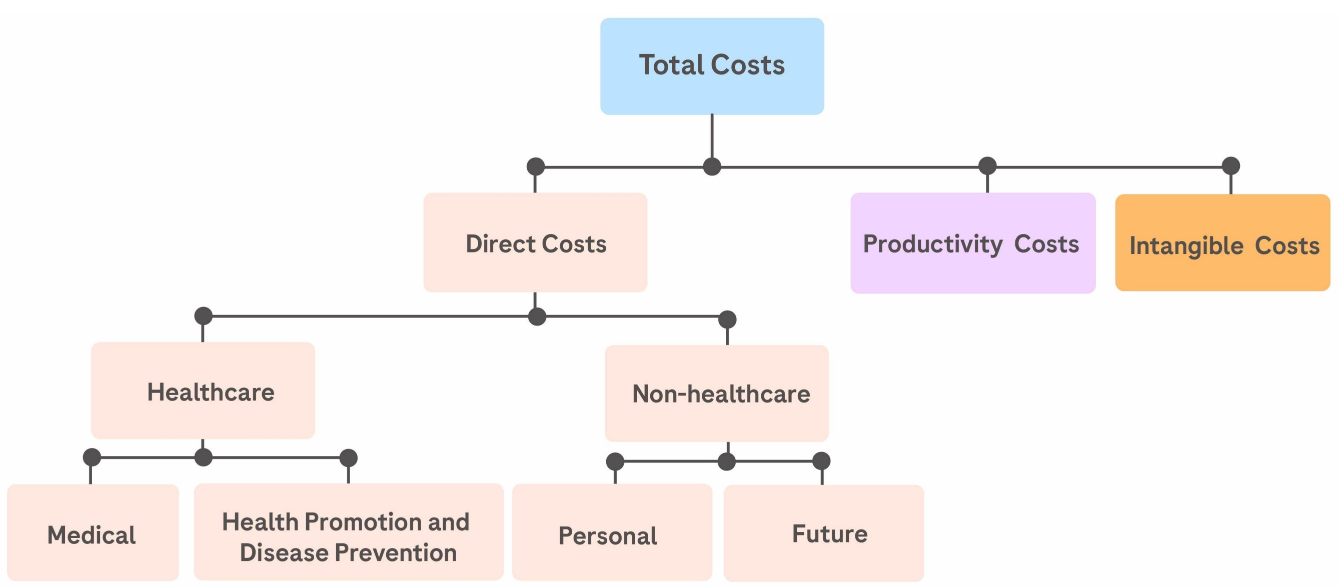

Figure S1. Cost Typology: disentangling health-related costs
